# A Mosaic Mutation in the *LAMA2* Gene in a Case of Merosin-deficient Congenital Muscular Dystrophy

**DOI:** 10.3389/fgene.2021.686800

**Published:** 2021-10-29

**Authors:** P. A. Chausova, O. P. Ryzhkova, G. E. Rudenskaya, V. B. Chernykh, O. A. Shchagina, A. V. Polyakov

**Affiliations:** Research Centre for Medical Genetics named after academician N.P. Bochkov, Ministry of Education and Science of the Russian Federation, Moscow, Russia

**Keywords:** congenital muscular dystrophy, molecular diagnostics, merosine deficient congenital muscular dystrophy, LAMA2, merosine, mosaicism, MPS

## Abstract

Merosine deficient congenital muscular dystrophy is one of the most common forms of congenital muscular dystrophy. This disease is caused by a primary deficiency or a functionally inactive form of the protein merosin in muscle tissue. The type of inheritance of this disease is autosomal recessive. *De novo* variants with this type of inheritance are rare, and it is quite possible that the *de novo* variant may hide a mosaic form in the parent of an affected child. We present a birth family with two affected children who inherited a previously undescribed pathogenic variant c.1755del from their mother and a previously described pathogenic variant c.9253C > T in the *LAMA2* gene from their mosaic father. *LAMA2* gene mutation analysis was performed by mass parallel sequencing and direct sequencing of genomic DNAs.

## Introduction

Congenital muscular dystrophies (CMDs) are clinically and genetically heterogeneous hereditary neuromuscular disorders, usually with autosomal recessive inheritance. CMDs are characterised by early manifestation, hypotonia, muscular weakness, contractures, and increased or normal creatine phosphokinase (CPK) levels. Electroneuromyography (ENMG) shows primary muscle lesions, and histological examination of muscle biopsy shows the dystrophic changes. In some cases, the central nervous system is affected, which leads to seizures and varying degrees of intellectual disability. Some patients die during infancy, others may live to adulthood (Dimova and Kremensky, 2018).

One of the most common CMD types is merosin-deficient congenital muscular dystrophy (OMIM 607855: MDC1A), which makes up to 36.4 and 37.4% of all CMD cases in China and UK respectively (Norwood et al., 2009; Sframeli et al., 2017; Ge et al., 2019). MDC1A is caused by primary merosin deficiency in muscle tissue, has autosomal recessive (AR) inheritance type, and is a result of mutations in the *LAMA2* gene (LAMININ, ALPHA-2), which is located on the long shoulder of chromosome 6 (locus 6q22.33) and comprised of 65 exons. *LAMA2* encodes the α2 laminin subunit of the heterotrimeric extracellular protein laminin-211 (laminin 2), which also includes subunits β1 (LAMB1) and γ1 (LAMC1). The LAMA2 protein consists of 3110 amino acid residues and includes six domains. The C-terminal G domain (encoded by exons 47–65) consists of 957 amino acid residues and binds to α-dystroglycan and integrin α-7/β-1 (Leiden Muscular Dystrophy, 2020).

To date, 406 pathogenis and likely pathogenic *LAMA2* variants have been described, including 41.1% missense and nonsense mutations, 18.5% splice mutations, 21.2% small deletions, 7.4% small insertions, 0.2% small indels, 8.6% gross deletions, 2.0% gross insertions, and 1.0% complex rearrangements (Human Gene Mutation, (2020)). Most of these variants are loss-of-function (LoF) (Oliveira et al., 2018), which often cause a severe form of the disorder. In these cases, immunohistochemical examination reveals a complete absence of merosin in muscle tissue. Missense variants usually cause partial merosin deficiency and a milder CMD form (Oliveira et al., 2018). At the same time, in-frame deletions and missense variants affecting the G domain lead to severe MDC1A, in spite of partial merosin presence in the basal membrane. This demonstrates the essential role of the G domain in binding laminin isoforms with integrins and α-dystroglycan (Bönnemann et al., 2014).

MDC1A is manifested in the postnatal period or infancy and is characterized by muscular weakness, hypotonia, and motor development delay. Joint contractures, scoliosis, and spine rigidity develop quickly. In rare cases, seizures and intellectual disability can be found. Severe complications include feeding difficulties (weak suction/chewing, intestinal peristalsis disfunction, dental pathologies, etc.) and respiratory distress. Independent movement is achieved rarely (Dadali et al., 2010; Chausova et al., 2020). Important criteria are white brain matter anomalies detected by MRI. Muscle biopsy shows signs of dystrophy, histochemical examination reveals absense or partial deficiency of merosin. Electroneuromyography (ENMG) detects primary muscular lesions, laboratory evaluation shows increased creatine phosphokinase (CPK) levels.

## Family Characteristics and Examination Methods

**FIGURE 1:**
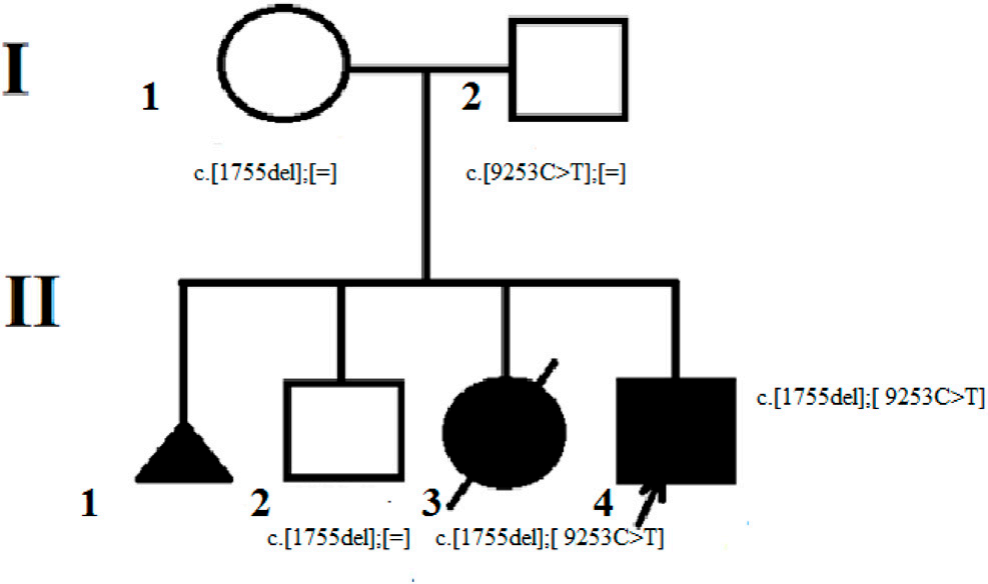
The proband’s family pedigree. The mother of the proband (I-1) is the carrier of the c. 1755del variant. The father of the proband (I – 2) is the carrier of c. 9253C> T variant in mosaic form. The brother of the proband (II-2) is also the carrier of c. 1755del variant. The proband (II-4) and his deceased sister (II-3) were found to have two pathogenic variants с. 1755del and c. 9253C> T.

The subject of this research is a Russian non-inbred family from Karelia (Figure 1). The proband was examined in the Research and Counseling Department of the Research Centre for Medical Genetics (RCMG) at the age of 3.5 months, then in 10 years the family was consulted without him on the subject of reproductive risks for the healthy older son: the counselor received medical documents of the proband and his deceased sister, as well as biological materials (blood samples of the proband, parents, brother and his wife; the sister’s paraffin-embedded muscle tissue samples; the father’s buccal epithelium cells and semen).

Molecular genetic alanysis was carried out for genome DNA samples extracted from peripheral blood lymphocytes, paraffin-embedded muscle tissue biopsy, buccal epithelium cells, and semen. The samples were analyzed *via* mass parallel sequencing (MPS) on a new-generation Ion S5™ sequencer (Thermo Scientific). The libraries prepared with ultramultiplex PCR were used for sequencing (AmpliSeq™). The “Congenital muscular dystrophy” target panel based on Ion Ampliseq technology included the following genes: *SELENON, LMNA, TPM3, ACTA1, PLOD1, MYPN, ITGA7, STAC3, CNTN1, MYF6, CFL2, KBTBD13, TRIP4, CHST14, CCDC78, MYH2, FKRP, TNNT1, RYR1, DNM2, COL6A3, KLHL41, BIN1, ZAK, SPEG, COL5A2, COL6A1, COL6A2, CHKB, ITGA9, KLHL40, LMOD3, MTMR14, MEGF10, LAMA2, COL12A1, DSE, FKBP14, AEBP1, TPM2, FKTN, COL5A1, MTM1,* and *VMA21*. The coverage of target variants for DNA extracted from blood lymphocytes was at least x350, from semen x654, from buccal epithelium x68. The detected variants were validated *via* Sanger sequencing. The detected variants were analyzed using a hg19 genome assembly, HGMD Professional Database v2020.2 (Human Gene Mutation, (2020)), and Mass Parallel Sequencing data interpretation guidelines (Ryzhkova et al., 2019).

## Results

### Clinical Data

The clinical data is based on the results of the proband’s early evaluation in RCMG, the children’s medical documents and the mother’s account.

The girl had uncomplicated pre- and perinatal medical history, but since birth had noticeable motor development delay, muscular hypotonia, areflexia. Around the age of 6 months multiple flexion contractures appeared in major joints (knees, hips, ankles, elbows) and quickly grew, later scoliosis became noticeable. She started holding her head with delay, sat on a chair if seated, could not sit up on her own, crawled on her buttocks, had no other motor skills. With time diffusal muscular atrophy progressed. Psychological and speech development corresponded to her age with the exception of moderate dysarthria. Face was normal, according to the photograph. ENMG showed lesions on muscular level. CPK levels were not measured. MRI showed combined hydrocephaly, atrophic changes of temporal lobes, no signs of white brain matter lesions. At the age of 3 years she was examined in the Russian Children’s Clinical Hospital, diagnosed with congenital nemaline myopathy (without biopsy). At the age of 5 years an operation was carried out to correct contractures (subspinal myotonia of hip flexors, hamstring and achilles tendon elongation). During early postoperative period (a few hours after emergence from anesthesia) there was aspiration of gastric contents with subsequent death. The aspiration detected *via* autopsy was reported as the direct cause of death. Histological examination of muscle tissue revealed profound atrophy, sclerosis, lipomatosis of intermuscular stroma. Specific nemaline staining was not used, but considering the clinical findings (brain tissue oedema, moderate enlargement of lateral ventricles), nemaline myopathy was probable.

The proband was born 7 years after his sister’s death. The parents knew that the daughter’s disorder was hereditary, but received incorrect information about high risk for female offspring only. The 41-year-old mother’s pregnancy was complicated by varicosity, chronic herpes infection, swelling and polyhydramnios in third trimester. Chorionic villi biopsy taken due to high risk of aneuploidy showed a 46, XY caryotype. The proband was born at 40 weeks with a mass of 3490 g, height 54 cm, Apgar score 6/7, and pronounced muscular hypotonia. Family history allowed to suggest congenital nemaline myopathy. His development was similar to his sister’s, though the contractures became noticeable earlier, at the age of 3 months, as well as *pectus excavatum*. The diagnosis surely was AR congenital myopathy. Further parenthood was not considered, and the parents, aware of non-specific treatment of multiple myopathy forms, did not consent to muscular biopsy or DNA diagnostics, which in 2009 was less developed. The proband was under observation in the Republican Children’s Hospital (Petrozavodsk, Russia). Brain MRI and CPK level measurement were not carried out. As well as his sister, he could hold his head, sat if seated, crawled on his buttocks, could move his hands, ate without help. Mental development was normal. At the age of 7 he had severe acute infective allergic Guillain–Barré syndrome (Landry’s paralysis); during treatment he was put on lung ventilation, required tracheostoma and gastrostoma. After this condition, he became bedridden. At present, he is 11.5 years old, breathes through tracheostoma for about 5 years with periodical lung ventilation, which leads to impaired speech: whisper with the tracheostoma and anarthria during ventilation, and eats through gastrostoma. He studies at home, plays complex video games, and so on. According to the medical data and photos from 2019, he had cachexia, severe flexion contractures of major joints, normal cranial innervation, severe diffuse tetraparesis with amyotrophy, normal wrist movement and partial feet movement, normal sensitivity; muscle tonus was difficult to evaluate due to the contractures, reflexes decreased/absent, abdominal reflexes absent.

Two practically identical cases in children of different sex born to healthy parents indicate an AR congenital structural myopathy or CMD, which means the initial diagnosis was incorrect. The disorder type was determined *via* DNA analysis.

### Molecular Genetic Analysis

The family was initially consulted on the subject of the older son’s reproductive risks, and despite the fact that in case of an AR disorder the risk is minimal, establishing the form of the disease, the type of its inheritance and determining the carriers of pathogenic variants in the family is an important aspect of medical and genetic counseling. However, specific methods of treatment for severe disorders earlier presumed incurable (including neuromuscular) are constantly being developed, therefore determining the CMD type is also essential.

Because the parents were sure of the nemaline myopathy (a genetically heterogeneous type) diagnosis, the RCMG DNA diagnostics laboratory analyzed the *ACTA1* gene as one of the causative genes*.* The analysis of the proband’s genomic DNA *via* Sanger sequencing did not detect any pathogenic or likely pathogenic variants in this gene.

The CMD target panel, which also includes genes that cause nemaline myopathy, was analyzed with MPS. It revealed two variants in the *LAMA2* gene (NM_000426.3): a c.9253C>T variant and a c.1755del variant. The c.9253C>T variant is located in exon 65, which leads to a premature translation termination site formation (p.(Arg3085Ter)). According to gnomAD v.2.1.1 (The Genome Aggregation Database) (Genome aggregation database, 2020), the frequency of this allele is 0.0003980% (1 allele in 251280). The prediction programs MutationTaster, LRT, fathmm_MKL_coding regard this variant as pathogenic. Accordingly, this variant, according to the ACMG classification, is pathogenic (PVS1. PM2. PM1. PM3. PP3. PP4) (Ryzhkova et al., 2019). Depth of coverage for this variant was x528.

**FIGURE 2:**
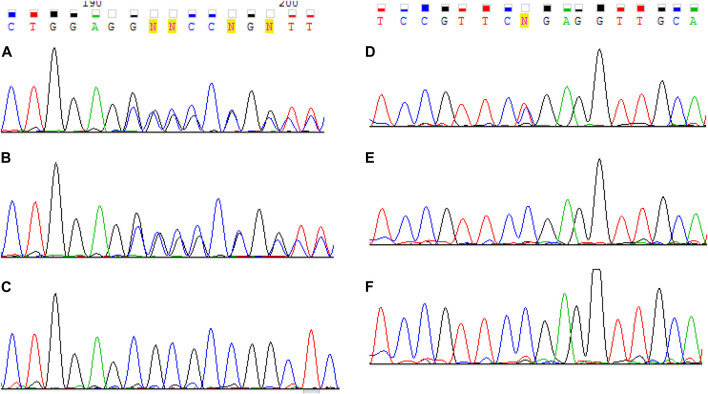
Sanger sequencing results of *LAMA2* exon 12 for the proband **(A)**, his mother **(B)** and father **(C)** and exon 65 for the proband **(D)**, his mother **(E)** and father **(F)**. The proband were found to have two pathogenic variants с. 1755del and c. 9253C> T. One pathogenic variant с 1755del was identified in the mother. The father is presumed to have the pathogenic c.9253C>T variant in mosaic form.

**FIGURE 3:**
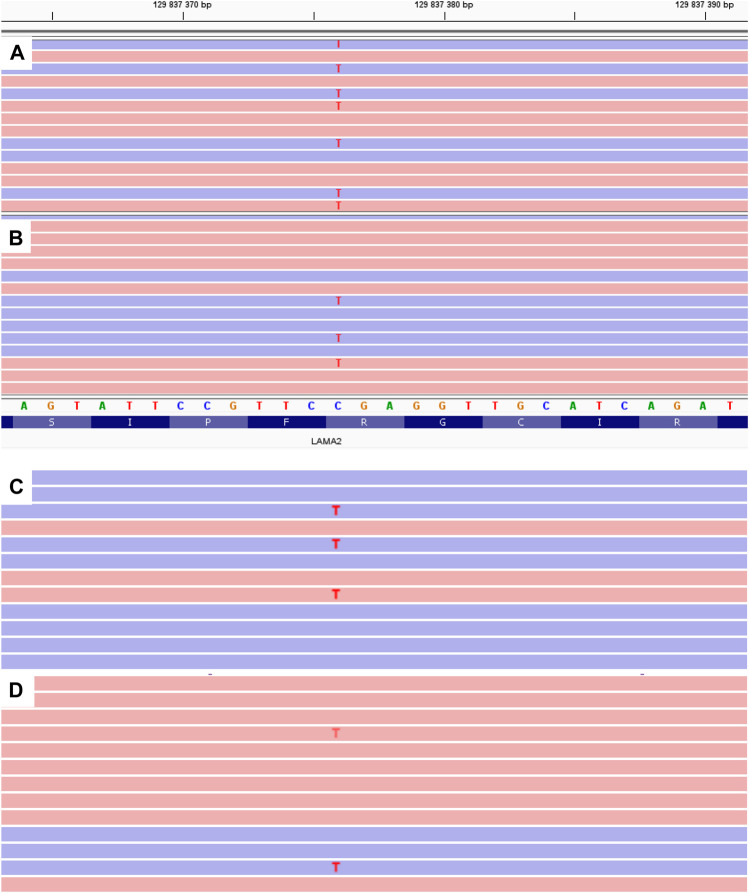
MPS results of *LAMA2* exon 65 for the proband **(A)** and his father **(B**–**D)**. B - genomic DNA extracted from peripheral blood lymphocytes. C - genomic DNA extracted from the buccal epithelium. C - genomic DNA extracted from the father’s semen.

A c.1755del variant is located in exon 12, previously not described as pathogenic, which leads to a frameshift and a premature translation termination site formation (p.(Ser585Argfs*14)). Depth of coverage for this point was x396. The variant is not registered in gnomAD v.2.1.1 (Genome aggregation database, 2020). Accordingly, this variant according to the ACMG classification is pathogenic (PVS1. PM2. PM3. PP4) (Ryzhkova et al., 2019). Samples of the parents’ and older brother’s genomic DNA extracted from peripheral blood lymphocytes were also analyzed with MPS. The patient’s mother and brother had the pathogenic c.1755del variant in heterozygous state (depth of coverage was x781 and 872 respectively). However, no pathogenic variants were detected in the father’s sample. A DNA test proved that the mother and father were the proband’s biological parents. Then all family members’ genomic DNA (extracted from peripheral blood lymphocytes or paraffin-embedded muscle tissue in case of the proband’s deceased sister) was scanned for target variants with Sanger sequencing. The c.1755del variant was detected in the proband’s mother and brother in heterozygous state, and the proband and his deceased sister had the c.1755del and с.9253C>T pathogenic variants in compound heterozygous state in non-mosaic form. Considering the acquired data, we suggested that the father had the latter variant in mosaic form (Figure 2), but it was impossible to determine with certainty whether the T fluorescent peak represented the mutant allele or background noise. Then we re-analyzed the father’s MPS data at the chr6:129837376 coordinate, which corresponds to c.9253C>T, and found the variant in mosaic form on 7% of all reads (24 out of 353) (Figure 3). The mutant clone prevalence in peripheral blood lymphocytes was presumed to be 14%. Further analysis using the MPS method of genomic DNA extracted from the father’s semen and buccal epithelium also showed the c.9253C>T variant in mosaic form (semen - 8%, 53 reads out of 654, buccal epithelium - 7%, five reads out of 68). The mutant clone prevalence is 8 and 14% respectively.

## Discussion

Seeing that the father had the mutation in mosaic form in all analyzed cells - mesodermal (lymphocytes), ectodermal (buccal epithelium), and spermatozoa - in approximately equal amounts, and considering the percentage of mutation carrying cells, we can assume that the mutation was already present in the blastocyst’s internal cellular mass. Germ layers and precursors of primary germline cells are developed from it, therefore the mutation has likely occurred before blastocyst formation, on the cleavage stage.

As shown in this study, MPS allows us to detect a variant in mosaic form that could be missed with Sanger sequencing. Thus, it can distinguish a *de novo* variant from a mosaic variant, granting important data for future reproduction planning. In case of a *de novo* variant, the reproductive risk is equal to the population risk, and in this case it was around 4% because of the mosaic pathogenic variant in the father.

Mosaic cases of collagenopathy (Donkervoort et al., 2015), α-dystroglycanopathy (*FKRP* gene) (Brockington et al., 2001), and congenital laminopathy (Makri et al., 2009) were described in literature, but MDC1A was not mentioned in this context. Although there are studies that describe *de novo* variants in the *LAMA2* gene (Tran et al., 2020). *De novo* variants are very rare for AR inheritance, and it could actually be a mosaic form in the patient’s parent. As shown above, without MPS this could be overlooked.

Indeed, in monogenic forms of diseases with an AR type of inheritance (as in MDC1A), the risk of having an affected child is 25%. In the presented case, due to the mosaic carriage of one of the paternal mutations, the calculated risk of having an affected child was 4%. However, this risk can be realized and does not decrease with each subsequent pregnancy. We believe that the influence of the “paternal” mutation on the preferential fertilization by spermatozoa “carrying” the pathogenic variant is unlikely. It is also unlikely that the mutation in children arose *de novo*, and was not inherited from the father. Thus, we consider the realization of the trait in two children in this family, albeit unfavorable, but an accident.

The c.1755del variant is LoF and is located in exon 12, which encodes the IVb merosin domain. It leads to a frameshift and a premature translation termination site formation (p.(Ser585Argfs*14)).

The c.9253C > T variant is a variant resulting in the shortening of the protein length. It is located in the exon closest to the 3’ region (exon 65), and causes the termination of the synthesis of amino acids in the LG5 domain, which is important for the binding of merosin to alpha-dystroglycan. Mutations in this domain result in disruption of the binding of merosin to alpha-dystroglycan, and cause CMD or LGMD. According to gnomAD v.2.1.1 (Genome aggregation database, 2020), the frequency of this allele is 0.0003980% (1 allele in 251280). The prediction programs MutationTaster, LRT, fathmm_MKL_coding regard this variant as pathogenic. Due to the fact that the programs for determining the pathogenicity of splice site mutations splice_ai, mmsplice do not predict the effect of substitution c.9253C > T on processing, the pathogenetic mechanism of its pathogenicity is precisely the premature termination of translation.

Unfortunately, in the described case it was impossible to obtain muscle biopsy results or the fibroblast test, which would have shown full or partial merosin absence. But the fact that this variant was described by other authors in patients with a phenotype similar to a pathogenic one (He et al., 2001; O'Grady et al., 2016), as well as the available description of another stop codon as pathogenic located closer to the 3’ region of the gene relative to the discussed varian (Capalbo et al., 2019), is, in our opinion, convincing evidence of its pathogenicity. Also in one of the published articles (O'Grady et al., 2016) a decrease in the amount of merosin in muscle tissue in a patient with a mutation with c.9253C>T was reported.

Most described variants causing severe CMD1A are loss-of-function in homozygous or compound heterozygous state, and this case contributes to the data.

## Data Availability

The datasets for this article are not publicly available due to concerns regarding participant/patient anonymity. Requests to access the datasets should be directed to the corresponding author.
